# Effects of fungal supplementation on endurance, immune function, and hematological profiles in adult athletes: a systematic review and meta-analysis

**DOI:** 10.3389/fnut.2025.1670416

**Published:** 2025-11-06

**Authors:** Meng-Yuan Shu, Xiu-Chang Zhang, Li Zuo, Fang-Lin Jiang, Jian Liang, Fang Li

**Affiliations:** 1 Experimental Teaching Demonstration Center of Food Safety and Nutrition, Xinjiang Institute of Technology, Aksu, China; 2 Aksu Institute of Apple, Xinjiang Institute of Technology, Aksu, China; 3 Department of Sports Medicine, Soonchunhyang University, Asan, Republic of Korea; 4 School of Sports Science, Hengyang Normal University, Hengyang, China; 5 School of Physical Education, Hunan Normal University, Changsha, China; 6 Department of Food Science and Engineering, Xinjiang Institute of Technology, Aksu, China; 7 Department of Biology, Soonchunhyang University, Asan, Republic of Korea; 8 School of Pharmacy, Jiangsu Medical College, Yancheng, China; 9 Department of Cancer Biology and Comprehensive Cancer Center, Wake Forest University School of Medicine, Winston Salem, NC, United States

**Keywords:** edible fungi, mushroom, Lingzhi, *Ganoderma lucidum*, athlete, sports performance

## Abstract

Edible fungi are rich in a wide array of bioactive compounds, and a growing body of evidence suggests that they possess anti-inflammatory, antioxidant, antitumor, anti-aging, and other beneficial properties. Although an increasing number of studies have investigated the potential benefits of fungal supplements for athletes, there is still a lack of comprehensive reviews that systematically synthesize the available evidence. We conducted a systematic literature search using the PubMed, Scopus, Web of Science, and CNKI databases. The search employed a combination of keywords, including mushroom, *Cordyceps*, *Ophiocordyceps sinensis*, Lion’s Mane mushroom, etc. and athlete. Fourteen randomized controlled trials involving 528 athletes were included, of which eight studies (*n* = 288) met the criteria for quantitative synthesis. Meta-analysis showed that *Cordyceps sinensis* supplementation significantly improved endurance performance (*p* = 0.05), ventilatory threshold (*p* = 0.03), and VO_2_peak (*p* = 0.04), indicating enhanced aerobic capacity with low heterogeneity. In contrast, *Ganoderma lucidum* supplementation resulted in significant reductions in blood urea nitrogen (*p* < 0.00001) and blood lactate (*p* < 0.00001), along with increases in hematocrit (*p* < 0.00001) and superoxide dismutase activity (*p* = 0.01). Subgroup analyses further revealed that both triterpenoid and polysaccharide extracts of *G. lucidum* significantly elevated hemoglobin concentrations (*p* < 0.00001), with stronger effects observed in endurance athletes. Our findings support the potential of fungal supplements as natural, safe, and effective ergogenic aids that enhance endurance, recovery, and physiological resilience in athletes.

## 1 Introduction

Fungi are ubiquitous in the natural world and across both artificial and natural habitats (1), playing vital roles in biogeochemical cycles, maintaining ecosystem balance, and influencing various aspects of human life (2). Although some fungal species can cause human health problems by inducing infections or producing mycotoxins, others such as yeasts and edible mushrooms are well known for their beneficial effects (3). Yeasts are essential for food fermentation and a variety of biotechnological processes, while edible fungi are valued for their nutritional content and potential medicinal properties (4).

Edible fungi are a group of macrofungi that encompass more than 2,000 identified species. More than 20 species have been successfully developed for large-scale commercial cultivation (5). Edible fungi are rich in a diverse array of bioactive compounds, including polysaccharides, alkaloids, sterols, polyphenols, terpenoids, peptides, and micronutrients such as vitamins and trace elements (6, 7). Numerous studies have highlighted their antioxidant properties (8), as well as their roles in cancer prevention and inhibition of tumor growth (9), anti-aging mechanisms (10), and lipid-lowering effects (11). In addition, certain components found in edible fungi have shown promise in regulating blood glucose and blood pressure levels (12, 13).

Athletes typically engage in prolonged periods of high-intensity physical activity, placing increased demands on their energy supply and metabolism, which in turn may induce oxidative stress, inflammation, and minor muscle injuries, ultimately affecting their training quality and competitive performance (14–16). Additionally, repeated intense training over extended periods may compromise the immune function, making athletes more susceptible to illnesses (17). Therefore, beyond basic nutritional intake, athletes require supplementary nutritional strategies to enhance antioxidant capacity, reduce fatigue, accelerate post-exercise recovery, and support immune health (18, 19).

Natural dietary supplements, especially those based on edible fungi, have recently gained considerable interest in sports nutrition. However, existing research has predominantly focused on the isolated effects of single types of edible fungi or specific bioactive compounds derived from these fungi rather than systematically evaluating their comprehensive physiological benefits (20). To date, there is a notable lack of systematic reviews that have comprehensively assessed the effects of edible fungi supplementation on athletic performance, endurance, fatigue mitigation, immune response, and recovery processes. To address this gap, the present systematic review aimed to critically analyze and synthesize the available literature examining the multifaceted effects of various edible fungi and their extracts on athletes. Through a comprehensive assessment, this review intends to provide theoretical insights and practical recommendations, thereby supporting future research directions and optimizing strategies for sports nutrition.

## 2 Materials and methods

### 2.1 Research design

This systematic review and meta-analysis were conducted using the Preferred Reporting Items for Systematic Reviews and Meta-Analyses (PRISMA) guidelines and established methodological standards (21). The protocol was registered in the PROSPERO database (registration no.: CRD420251035783). This review aimed to examine the effects of fungal supplementation on the endurance, immune function, and hematological parameters of athletes. By integrating findings from existing studies, this study sought to clarify the potential benefits of such supplements in sports performance and recovery, while addressing important gaps in the current body of evidence.

### 2.2 Search strategy and inclusion criteria

A comprehensive literature search was conducted across three major databases (PubMed, Scopus, and Web of Science) using the following keywords: mushroom, *Cordyceps*, Ophiocordyceps sinensis, Lion’s Mane mushroom, *Hericium erinaceus*, wood ear, *Auricularia auricula*, *Auricularia heimuer*, *Auricularia cornea*, *Tremella fuciformis*, *Ganoderma lucidum*, Reishi, Lingzhi, *Coriolus versicolor*, mushroom polysaccharides, *Pleurotus ostreatus*, oyster mushroom, Maitake, *Grifola frondosa*, Shiitake, *Lentinula edodes*, and *Phellinus linteus*, in combination with the term athlete. The search was independently performed by two reviewers (M-Y.S. and J.L.) on April 1, 2025, and was initially limited to studies published in English.

To minimize potential language and regional bias, additional searches were conducted in China National Knowledge Infrastructure (CNKI) and Japan Science and Technology Information Aggregator, Electronic (J-STAGE). For CNKI, relevant Chinese-language studies were retrieved and reviewed by native speakers. Moreover, the CNKI search specifically targeted articles indexed in the Peking University Core Journals database to ensure the inclusion of high-quality, peer-reviewed research published in reputable Chinese journals. An attempt was also made to search J-STAGE using machine translation tools (Google Translate) to translate keywords, titles, and abstracts into English. However, the translation quality was insufficient for accurate interpretation of study design and outcomes, posing a risk of data misclassification. Therefore, only Chinese-language articles meeting the inclusion criteria were incorporated, while Japanese-language records were excluded to maintain methodological reliability. The detailed multilingual search strategies used for CNKI and J-STAGE are presented in Supplementary Table S2.

The inclusion criteria were as follows: (1) randomized controlled trials (RCTs) involving at least one intervention group receiving a fungal extract or mushroom-based supplements and a placebo control group; (2) studies in which participants consumed only mushroom-based supplements (either a single species or a combination), without other concurrent supplements unless both groups received the same co-supplementation; (3) studies involving adult athletes; and (4) peer-reviewed literature only—gray literature and case reports were excluded.

### 2.3 Data extraction and outcomes

Two authors (M.Y.S and J.L.) independently identified and selected the studies. Data extraction was performed using a predefined Excel spreadsheet, which included the following variables: study information, type of fungal supplement, dosage, intervention duration, participant demographics (including sex), physical characteristics, athlete type, competitive level, and main outcomes. In cases where discrepancies arose in the extracted data, the two researchers collaboratively conducted a second round of data extraction to verify the accuracy of the information.

### 2.4 Risk of bias

The risk of bias in the included studies was assessed independently by two researchers using the Cochrane Risk of Bias Tool for Randomized Trials (RoB 2.0) (22). The assessment covered five domains: randomization process, deviations from intended interventions, missing outcome data, measurement of the outcome, and selection of the reported results (22). In cases of disagreement, a third researcher (X.C.Z.) was consulted to resolve any discrepancies.

### 2.5 Statistical analyses

Statistical analyses were conducted using Review Manager version 5.4. Standardized mean differences or mean differences were calculated for all included studies. Forest plots were generated to visually display heterogeneity among the studies.

## 3 Results

### 3.1 Search results

**Figure 1:**
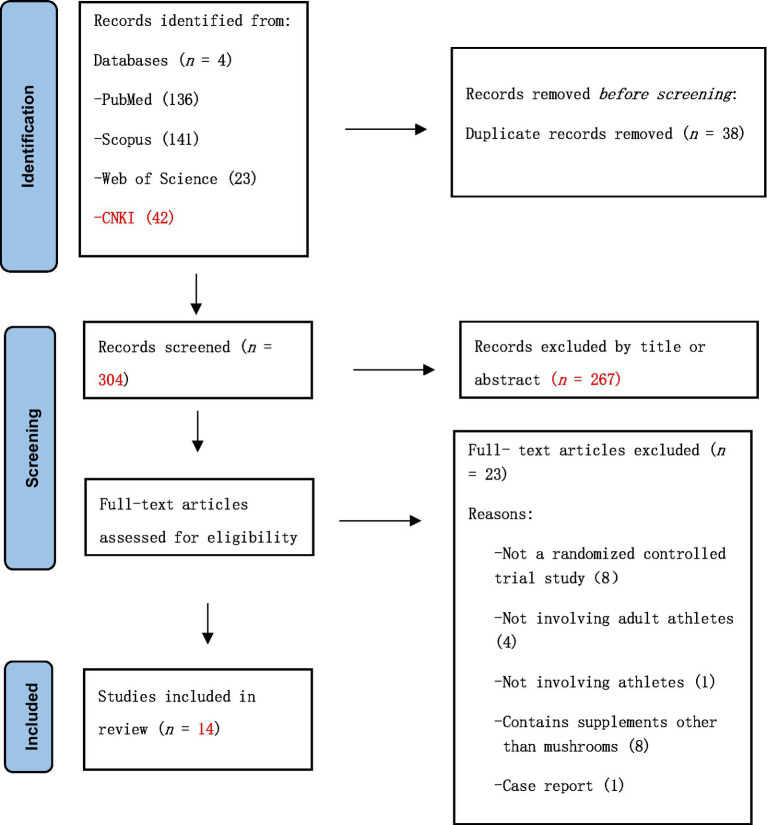
Flow chart for study inclusion and exclusion process based on PRISMA.

In total, 342 records were identified through database searches. After excluding 38 duplicates, 304 records remained. After screening the titles and abstracts, 267 articles were excluded. The full texts of 37 articles were assessed for eligibility. Of these, 23 were excluded for the following reasons: not a randomized controlled trial (*n* = 8), not involving adult athletes (*n* = 4), not involving athletes (*n* = 1), included supplements other than mushrooms (*n* = 8), case reports (*n* = 1), and unavailable full text (*n* = 1). Finally, fourteen studies met the inclusion criteria and were included in this systematic review and meta-analysis. The PRISMA flow diagram shows the study selection process (Figure 1).

### 3.2 Characteristics of included studies

A total of fourteen randomized controlled trials (RCTs) published between 2004 and 2024 were included, involving 528 athletes who participated in endurance, team, and multi-sport disciplines. The participants were male and female athletes, including cyclists, football players, wrestlers, long-distance runners, basketball players, and winter-sport athletes. The duration of the interventions ranged from 2 weeks to 4 months (Table 1).

**Table 1:** Studies examining the characteristics associated with the use of fungal supplements in athletes.

Study	Fungal supplement	Dosage	Duration	Participants (sex)	Physical characteristics	Athletes’ type and level	Results
Parcell et al., 2004 (34)	*Cordyceps sinensis*	3 g/day (2 capsules, 3×/day)	5 Weeks	22 Males	*Cordyceps sinensis* group: 25 ± 5 years; 175 ± 12 cm; 73 ± 8 kg; Placebo group: 25 ± 3 years; 176 ± 6 cm; 69 ± 6 kg	Endurance-trained male cyclists	No significant effect on VO_2_peak, ventilatory threshold, or time trial performance compared with placebo.
Zhang et al., 2008 (49)	*Ganoderma lucidum*	LHTL1: Placebo (10 caps/day); LHTL2: 2.5 g/day = 10 caps/day (250 mg each); LHTL3: 5.0 g/day = 20 caps/day	6 Weeks total (2 weeks baseline supplementation; 4 weeks of LHTL protocol)	40 Males	Control group (mean): 21.4 years, 175.5 cm, 69.25 kg; LHTL1: 21.3 years, 175.9 cm, 68.75 kg; LHTL2: 21.1 years, 176.1 cm, 67.81 kg; LHTL3: 21.6 years, 176.1 cm, 69.44 kg.	Elite male football players from Beijing Sport University	LHTL training alone (Placebo group) significantly decreased CD4^+^/CD8^+^ ratio; 2.5 g/day (LHTL2) did not prevent decrease in CD4^+^/CD8^+^; 5.0 g/day (LHTL3) showed a trend toward improvement of CD4^+^/CD8^+^ ratio vs. LHTL1 & LHTL2, but not always significant; CD3^+^ T cell % increased significantly at day 21 in the LHTL3 group.
Bobovčák et al., 2010 (24)	insoluble β-glucan extracted from *Pleurotus ostreatus*	100 mg/day (pleuran + 100 mg Vitamin C); Placebo: 100 mg fructose + 100 mg Vitamin C	2 Months	16 Males, 4 females	Pleuran group: 23.6 ± 1.7 years, 174.7 ± 3.2 cm; Placebo group: 24.0 ± 0.9 years, 175.6 ± 2.4 cm.	Elite athletes from winter sports (cross-country skiing, figure skating, sledge sports).	After intensive exercise, both groups showed a significant increase in leukocyte, T cell, B cell, and monocyte counts (*p* < 0.001). However, during the recovery period, the Placebo group exhibited a significant 28% decrease in natural killer (NK) cell activity (NKCA) (*p* < 0.01) and a significant reduction in NK cell count (*p* < 0.05), whereas the β-glucan (Pleuran) group showed no significant decrease in either NKCA or NK cell number.
Bergendiova et al., 2011 (64)	Insoluble β-glucan extracted from *Pleurotus ostreatus*	200 mg/day (2 capsules containing 100 mg pleuran + 100 mg vitamin C)	3 Months	26 Males, 24 females	Pleuran group: 23.6 ± 0.8 years; Placebo group: 24.0 ± 0.9 years	Elite-level athletes from the National Sport Centre in various sports (canoeing, swimming, cycling, etc.)	After 3 months, the Pleuran group showed a significant reduction in URTI symptoms (*p* < 0.001), with fewer athletes reporting ≥4 symptoms (12% vs. 84%). NK cell count increased significantly in the Pleuran group and remained elevated at 6 months (*p* < 0.001). Phagocytosis remained stable in the Pleuran group, whereas the Placebo group showed a significant decline (*p* < 0.001), with a significant between-group difference (*p* < 0.01).
Liu, 2016 (26)	*Ganoderma lucidum* polysaccharide oral liquid	10 mL twice daily (after breakfast and dinner), taken for 6 days with 1 day off, continuously for 90 days	90 Days	11 Males and 9 females in experimental group; 12 males and 8 females in control group	Age: 21 ± 1.5 years; all had >1 year of training experience	University cycling team athletes under pre-competition training	Supplementation significantly improved total work output, load duration, and maximal exercise heart rate, while decreasing exercise heart rate and body fat percentage. Hemoglobin and hematocrit increased, lactate decreased (*p* < 0.05). Antioxidant enzyme activities (SOD, CAT) and glucose increased, while BUN decreased (*p* < 0.05). IgG, IgA, and IgM levels were elevated, and declines in Et and Ea were less pronounced than in controls (*p* < 0.05).
Du and Song, 2019 (29)	*Cordyceps sinensis* polysaccharide extract	10 mg·kg^−1^ body weight, taken orally 1 h before bedtime	3 Weeks (training 6 days/week, rest 1 day)	40 Female wrestlers	Age: 19–26 years; Height: 166.9–168.0 cm; Weight: ~65–66 kg; all healthy, no allergies or chronic diseases	Female wrestlers; high-intensity training; national first-level	Compared with control, supplementation significantly increased serum IgG and IgM (*p* < 0.05), with no significant change in IgA; complement components C3, C4, and CH50 activity significantly increased; reversed complement suppression induced by high-intensity training; demonstrated safe and non-stimulant nutritional enhancement.
Li, 2020 (55)	*Ganoderma lucidum* polysaccharide oral liquid	Polysaccharide oral liquid 10 mL twice daily (after breakfast and dinner, 6 days on/1 day off)	90 Days	60 University cyclists (male and female)	Age: 21 ± 1.5 years; trained ≥1 year	Collegiate cycling athletes	Increased hemoglobin and hematocrit, decreased lactate; elevated IgG, IgA, IgM; smaller decline in Et and Ea; improved endurance and immune recovery (*p* < 0.05).
Liu, 2020 (58)	*Ganoderma* spp. extract capsules (containing triterpenes and polysaccharides)	75 mg/capsule, 2 capsules per day (before breakfast)	8 Weeks	60 Male athletes (30 in supplement group, 30 in placebo group), aged 18–21 years	Aged 18–21 years	Collegiate-level athletes undergoing regular physical training	Ganoderma extract supplementation (150 mg/day) significantly increased grip strength (*p* < 0.05) and VO₂max (from 45.09 ± 9.88 to 51.40 ± 8.40 mL·kg^−1^·min^−1^, *p* < 0.05). No significant effect on flexibility or sprint speed. Blood biochemistry remained within normal range, with minor increases in Hb, BUN, and ALP, and decreases in creatinine levels. Suggests improved muscle strength and cardiopulmonary fitness with good safety profile.
Tang, 2020 (56)	*Ganoderma lucidum* triterpenoid capsules (GLTs)	75 mg per capsule, 1 capsule daily before morning training	30 Days	48 University athletes (24 males, 24 females) randomly divided into control and experimental groups	Age: 22.4 ± 1.3 yr.; weight: 62 ± 2.4 kg; 3–4 years of training experience; no organic disease	University-level athletes under regular daily training	Compared with control, GLT supplementation for 30 days significantly increased RBC (4.61 ± 0.21 × 10^12^ L^−1^), reticulocytes (1.04 ± 0.22%), hemoglobin (157.53 ± 8.66 g/L), and SOD (332.3 ± 16.58 U/L); and decreased HR (115.4 ± 3.45 bpm), serum lactate (13.68 ± 1.76 mmol/L), BUN (9.83 ± 1.95 mmol/L), AST (59.7 ± 7.82 U/L), and LDH (149.36 ± 15.69 U/L) (all *p* < 0.05). Indicates improved antioxidant defense and faster post-exercise recovery.
Wang, 2020 (76)	*Tremella fuciformis* polysaccharide oral liquid	Low = 10 mg/mL, Medium = 20 mg/mL, High = 30 mg/mL, 2.5 mL·kg^−1^ daily; placebo in control group	3 Weeks (2 weeks regular + 1 week HCT training)	60 Male basketball players (15 per group)	Age ≈ 19 years; Height ≈ 185 cm; Weight ≈ 66 kg; Training experience ≈ 7 years; Healthy, no smoking or alcohol habit	University basketball athletes (National Class II and above)	Supplementation significantly increased IgA, IgG, IgM levels (*p* < 0.05), reduced TNF-α and IL-10 levels (*p* < 0.05), and lowered fasting blood glucose (*p* < 0.05). High-dose group showed the most pronounced effect, indicating attenuation of exercise-induced immune suppression and improved glucose utilization.
Thongsawang et al., 2021 (23)	*Cordyceps sinensis*	3 g/day (CS powder mixed with stevia, once daily)	2 Weeks (with crossover design and 1-week washout period)	12 Males	Age: 37.3 ± 3.4 years; Height: 172.3 ± 5.2 cm	Trained long-distance runners (≥3 days/week running, >1 year)	Compared with placebo, CS significantly improved time to exhaustion, VO₂max, and VT2 (*p* < 0.05); no effect on VT1. CS effects were similar to sodium bicarbonate.
Zhao, 2021 (57)	*Ganoderma lucidum* polysaccharide tablets (250 mg per tablet)	Group 1: 250 mg twice daily; Group 2: 500 mg twice daily; Group 3: 750 mg twice daily; Control: placebo; duration of 30 days (10-day cycles)	30 Days	32 Male athletes (8 per group)	Age 18–20 years; body weight ≈ 62 kg; height ≈ 177 cm; 1–2 years training experience	Male collegiate athletes with National Class II certificate	Ganoderma polysaccharide supplementation significantly improved exercise work output and duration (*p* < 0.01), reduced blood lactate (*p* < 0.01), increased hemoglobin (*p* < 0.05), SOD and CAT activities (*p* < 0.01), and decreased lipid peroxide (LPO) levels (*p* < 0.01), demonstrating enhanced antioxidant and anti-fatigue effects.
Savioli et al., 2022 (25)	*Cordyceps sinensis*	2 g/day (667 mg x 3 times/day)	12 Weeks	13 Males, 9 females	CS group: 37.6 ± 6.4 years; Placebo group: 37.6 ± 7.7 years	Amateur marathoners, >8 years of experience, ≥300 min aerobic training/week	Improved 5 K time at week 12; no significant changes in Vo2max or ventilatory thresholds
Nakamura et al., 2024 (71)	*Cordyceps militaris* mycelium extract	1,800 mg/day (6 capsules/day)	16 Weeks	22 Males	CM group: 20.2 ± 1.1 years, 172.0 ± 5.4 cm, 57.2 ± 4.3 kg; Placebo group: 19.7 ± 0.9 years, 170.6 ± 6.6 cm, 56.6 ± 4.4 kg	University-level long-distance runners	The CM group showed a significant increase in serum ferritin levels at 4 and 8 weeks, higher hemoglobin and hematocrit change rates at 8 weeks, and a significant reduction in creatine kinase levels at 16 weeks compared with the Placebo group (all *p* < 0.05).

Among the investigated fungi, six randomized controlled trials examined *Ganoderma lucidum* and its polysaccharide or triterpenoid extracts, with dosages ranging from 75 mg to 5 g per day. Four randomized controlled trials investigated *Cordyceps sinensis*, administered at 2–3 g per day or 10 mg·kg^−1^ of body weight for 2–12 weeks. Two studies evaluated insoluble *β*-glucan (Pleuran) derived from *Pleurotus ostreatus*, at 100–200 mg per day for 2–3 months. In addition, one study using *Cordyceps militaris* mycelium extract (1,800 mg per day for 16 weeks) was conducted in university-level long-distance runners, and another investigated *Tremella fuciformis* polysaccharide oral liquid, administered for 3 weeks in male basketball athletes.

### 3.3 Assessment of risk of bias

As shown in Table 2, most studies exhibited a low risk of bias in the domains of missing outcome data and measurement of the outcome. However, some concerns were identified in other domains. One study (23) was judged to have a high risk of bias due to deviations from the intended interventions. Regarding the overall risk of bias, two studies (24, 25) were rated as low risk, one study (23) as high risk, and the remaining eleven studies were assessed as having some concerns.

**Table 2:** Domain-specific and overall risk of bias assessment for the included studies.

	Randomization process	Deviations from intended interventions	Missing outcome data	Measurement of the outcome	Selection of the reported result	Overall
Parcell et al., 2004 (34)	Low risk	Low risk	Low risk	Low risk	Some concerns	Some concerns
Zhang et al., 2008 (49)	Low risk	Some concerns	Low risk	Low risk	Low risk	Some concerns
Bobovčák et al., 2010 (24)	Low risk	Low risk	Low risk	Low risk	Low risk	Low risk
Bergendiova et al., 2011 (64)	Low risk	Low risk	Low risk	Some concerns	Some concerns	Some concerns
Liu, 2016 (26)	Some concerns	Some concerns	Low risk	Low risk	Some concerns	Some concerns
Du and Song, 2019 (29)	Some concerns	Low risk	Low risk	Low risk	Low risk	Some concerns
Li, 2020 (55)	Some concerns	Some concerns	Low risk	Low risk	Some concerns	Some concerns
Liu, 2020 (58)	Some concerns	Low risk	Low risk	Low risk	Some concerns	Some concerns
Tang, 2020 (56)	Some concerns	Low risk	Low risk	Low risk	Low risk	Some concerns
Wang, 2020 (76)	Some concerns	Low risk	Some concerns	Low risk	Some concerns	Some concerns
Thongsawang et al., 2021 (23)	Low risk	High risk	Low risk	Low risk	Low risk	High risk
Zhao et al., 2021 (57)	Some concerns	Low risk	Some concerns	Low risk	Some concerns	Some concerns
Savioli et al., 2022 (25)	Low risk	Low risk	Low risk	Low risk	Low risk	Low risk
Nakamura et al., 2024 (71)	Low risk	Low risk	Low risk	Low risk	Some concerns	Some concerns

### 3.4 Meta-analysis results

Among the 14 studies included in this review, eight met the criteria for meta-analysis, comprising a total of 288 athletes. These studies were selected because they shared comparable intervention characteristics, whereas the remaining studies were excluded due to incomplete numerical data or the use of different fungal species, making quantitative synthesis unfeasible.

**Figure 2:**
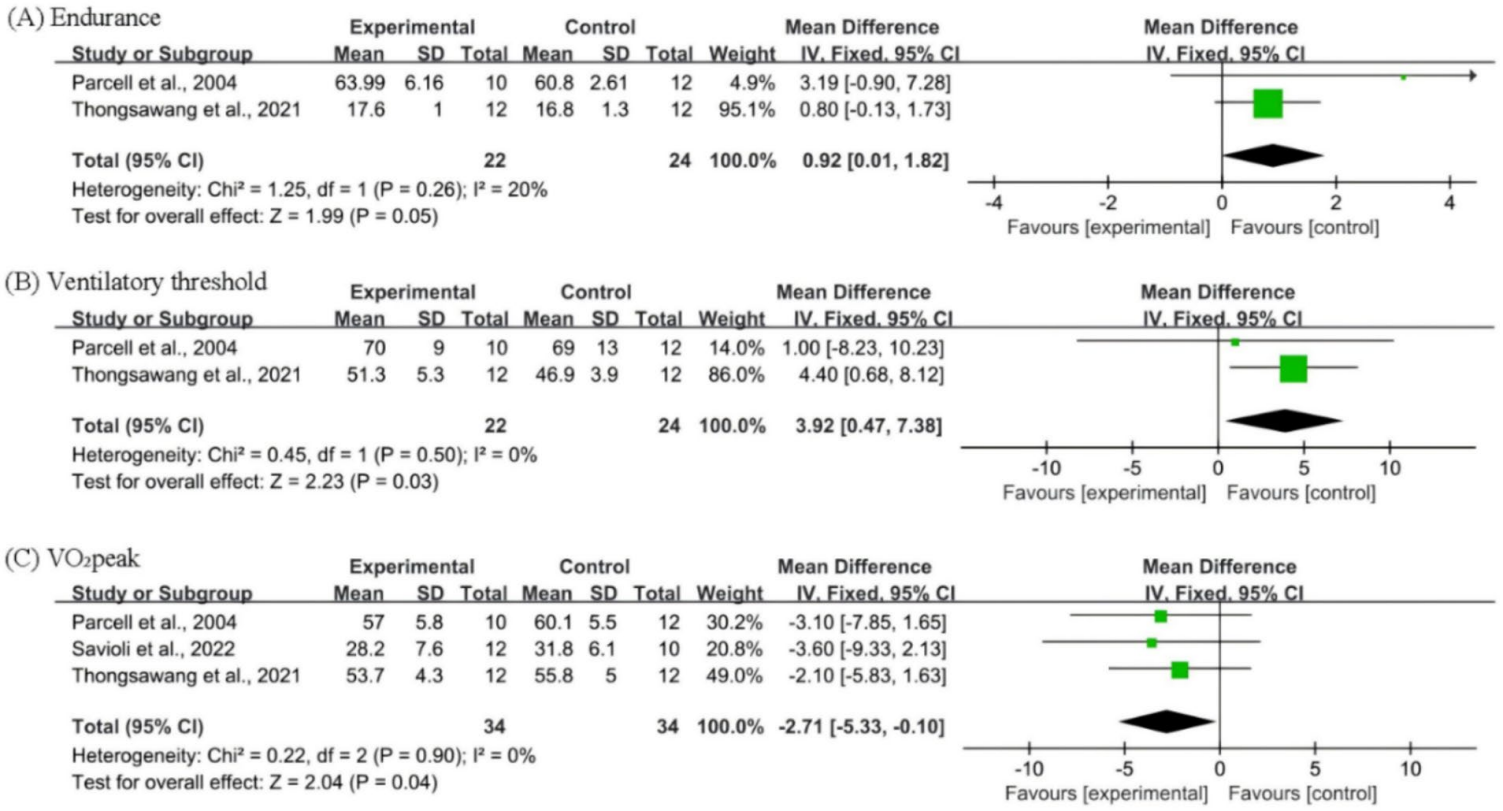
Forest plots for the *C. sinensis* supplementation group and the Placebo group. **(A)** Endurance; **(B)** Ventilatory threshold; **(C)** VO_2_ peak.

Forest plots were generated for three performance-related outcome measures, namely endurance, ventilatory threshold, and VO_2_peak, to evaluate the effects of *C. sinensis* supplementation on athletic performance (Figure 2). For endurance (Figure 2A), the pooled analysis of the two studies indicated a marginally significant improvement in the *C. sinensis* supplementation group compared with the placebo group (*p* = 0.05), with low heterogeneity (I^2^ = 20%). In terms of ventilatory threshold (Figure 2B), *C. sinensis* supplementation was associated with a statistically significant enhancement (*p* = 0.03) with no observed heterogeneity (I^2^ = 0%). A significant improvement was also noted in VO_2_peak (Figure 2C), favoring the *C. sinensis* group (*p* = 0.04), with negligible heterogeneity (I^2^ = 0%).

**Figure 3:**
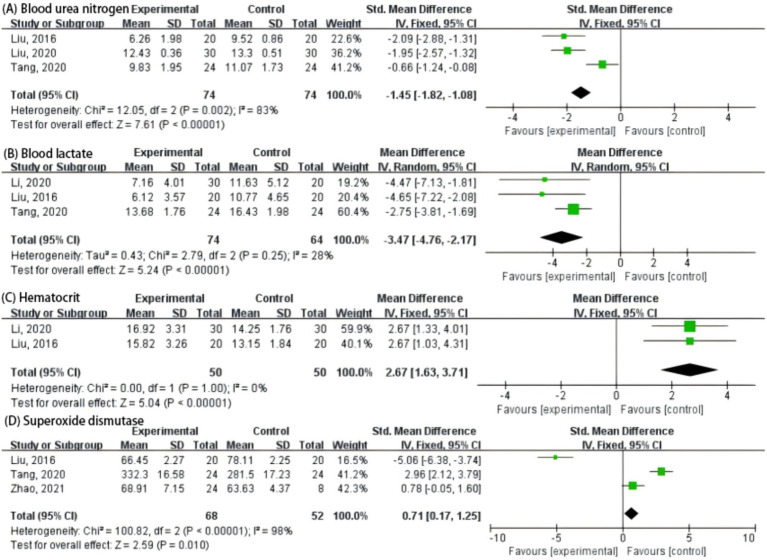
Forest plot of the effects of *Cordyceps* supplementation on endurance performance indicators in athletes. **(A)** Blood urea nitrogen; **(B)** Blood lactate; **(C)** Hematocrit; **(D)** Superoxide dismutase.

*Ganoderma lucidum* supplementation significantly influenced several hematological and biochemical parameters in athletes. Compared with the control, the experimental group showed a marked reduction in blood urea nitrogen (*p* < 0.00001) (Figure 3A) and blood lactate levels (*p* < 0.00001) (Figure 3B), indicating improved nitrogen metabolism and post-exercise recovery. Conversely, hematocrit values were significantly elevated following *G. lucidum* intake (*p* < 0.00001) (Figure 3C), suggesting enhanced erythropoietic capacity or oxygen-carrying potential. In addition, superoxide dismutase (SOD) activity increased modestly but significantly (*p* = 0.01), implying improved antioxidant defense (Figure 3D).

**Figure 4:**
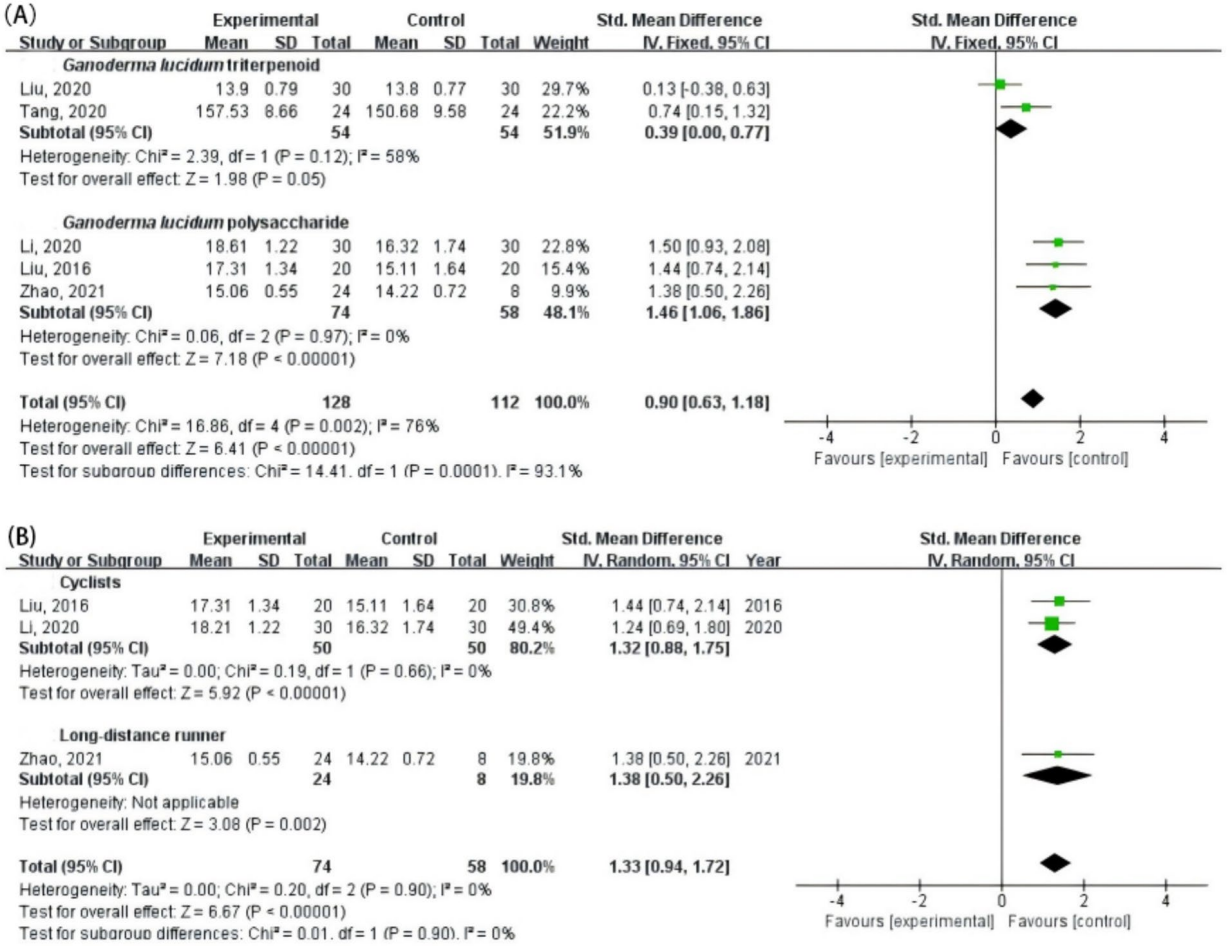
Subgroup analyses of the effects of *G. lucidum* supplementation on hemoglobin concentration in athletes. **(A)** By active compound type (triterpenoid vs. polysaccharide); **(B)** By athlete type (cyclists vs. long-distance runners).

As shown in Figure 4, subgroup analyses were conducted to examine the effects of *G. lucidum* supplementation on hemoglobin concentration across different extract types and athlete categories. In the subgroup analysis by extract type (Figure 4A), both triterpenoid and polysaccharide fractions significantly increased hemoglobin levels compared with control. The pooled effect size was larger for *G. lucidum* polysaccharides (*p* < 0.00001) than for triterpenoids (*p* = 0.05), and the difference between subgroups was statistically significant (*p* = 0.0001). In the athlete-type subgroup (Figure 4B), *G. lucidum* supplementation significantly improved hemoglobin concentration in both cyclists (*p* < 0.00001) and long-distance runners (*p* = 0.002), with no evidence of heterogeneity (I^2^ = 0%).

## 4 Discussion

### 4.1 Effect of fungal supplements in adult athletes

#### 4.1.1 Cordyceps sinensis

*Cordyceps sinensis* is widely recognized as one of East Asia’s most esteemed natural tonic herbs (26). It contains diverse bioactive compounds, including polysaccharides, cordycepin, and adenosine, which have been shown to activate the human immune system and exert a range of protective effects (27, 28).

In addition to its endurance-related benefits, recent evidence also highlights the immunomodulatory effects of *C. sinensis* polysaccharides in athletes engaged in high-intensity training. For example, Du and Song (29) reported that 3 weeks of oral supplementation (10 mg·kg^−1^·day^−1^) in national-level female wrestlers significantly increased serum IgG and IgM levels (*p* < 0.05), while complement components C3, C4, and CH50 activity were also elevated, effectively counteracting the complement suppression induced by intensive exercise. These findings suggest that *C. sinensis* polysaccharides can enhance immune resilience and recovery under high physical stress. Cordycepin (3′-deoxyadenosine) has been shown to activate steroid hormone production via adenosine receptor signaling in Leydig cells (30). In mice, cordycepin increased plasma testosterone by stimulating the cAMP/PKA pathway and upregulating the steroidogenic acute regulatory (StAR) protein. Elevated anabolic hormones can improve an athlete’s recovery, muscle maintenance, and red blood cell production, potentially explaining improved performance (30).

**Figure 5:**
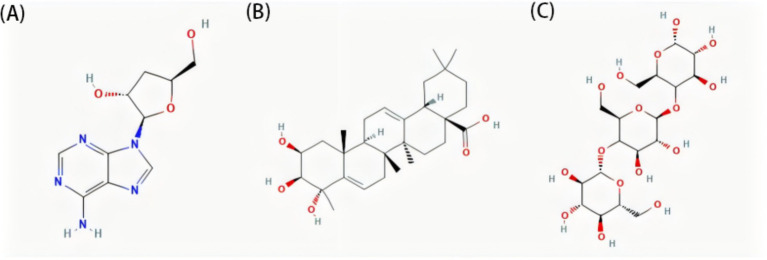
Representative bioactive compounds in edible fungi. **(A)** Cordycepin, **(B)** triterpenoid and **(C)** *β*-glucan structures. Chemical structures sourced from the PubChem database (https://pubchem.ncbi.nlm.nih.gov/).

Endurance sports are characterized by prolonged moderate-to-high-intensity physical activity that places considerable demand on aerobic metabolism (31). During these activities, adenosine triphosphate is primarily generated through oxidative phosphorylation, utilizing carbohydrates and fats as the main energy substrates (32). Therefore, efficient oxygen utilization, mitochondrial function, and fatigue resistance are critical determinants of endurance in athletic performance (33). Three studies evaluated the effects of *C. sinensis* supplementation (2–3 g/day) in endurance athletes (cyclists and runners) (23, 25, 34). These investigations assessed key physiological outcomes, such as aerobic capacity, fatigue resistance, and overall exercise performance, following supplementation. Among these, two studies demonstrated positive effects of *C. sinensis* supplementation on endurance performance, ventilatory threshold, and VO_2_peak in runners (23, 25), whereas one study involving cyclists reported no significant improvements (35). The divergent results observed across the studies may be attributed to two main factors. First, in the study in-volving cyclists, the *C. sinensis* used was commercially cultivated. Cordycepin, a key bio-active compound considered central to the physiological efficacy of the herb (35) (Figure 5A). Previous studies demonstrated that cordycepin stimulates steroidogenesis, which may contribute to improved physical performance (36). Mechanistically, cordycepin may further enhance aerobic metabolism by stimulating mitochondrial oxidative phosphorylation and promoting erythrocyte oxygen-carrying efficiency through increased 2,3-bisphosphoglycerate synthesis (37, 38). Second, differences in training characteristics between cyclists and long-distance runners, including variations in muscle recruitment patterns, training intensity, and energy metabolism, may influence physiological responsiveness to supplementation (39). Future studies should focus on the origin and composition of *C. sinensis* supplements, particularly regarding the presence of key active compounds such as cordycepin, which may play an important role in mediating physiological effects (40). Ensuring consistent quality and chemical characterization of the supplements used will help improve the comparability of findings across studies. In addition, differences in training background, physiological demands, and sports-specific characteristics should be considered, as these factors may influence individual responses to supplementation (41). Further research with larger sample sizes, longer intervention periods, and a clearer focus on the underlying mechanisms is necessary to confirm and better understand the potential benefits of *C. sinensis* supplementation in endurance athletes.

#### 4.1.2 Ganoderma lucidum

*Ganoderma lucidum*, similar to *C. sinensis*, has long been regarded in East Asia as a valuable traditional tonic (42), which is a rich source of structurally diverse bioactive constituents, with approximately 400 compounds identified to date, predominantly triterpenoids and polysaccharides (43, 44) (Figure 5B). These molecules are recognized for their broad spectrum of bio-logical functions, including modulation of inflammatory responses, enhancement of immune activity, antioxidative effects, antitumor actions, and antimicrobial properties (45–47). Prolonged exposure to hypoxic conditions combined with high-intensity training may disrupt T-lymphocyte homeostasis in football players (48). However, daily supple-mentation with 5 g of *G. lucidum* mitigates this imbalance by improving the CD4^+^/CD8^+^ ratio, whereas a lower dose of 2.5 g does not exhibit similar effects (49). Polysaccharides are the principal immunomodulatory constituents of *G. lucidum* and are known to enhance both innate and adaptive immune responses (50). They promote the proliferation and activation of key immune cells, including macrophages, T and B lymphocytes, natural killer (NK) cells, and lymphokine-activated killer cells. Polysaccharides from *G. lucidum* may promote T-regulatory-cell differentiation and modulate the CD4^+^/CD8^+^ ratio by activating dendritic-cell–dependent cytokine cascades (51), thereby potentially contributing to immune homeostasis under training stress. In addition, they stimulate splenocyte expansion and increase the production of cytokines and antibodies, thereby supporting a more coordinated and effective immune response (52, 53).

Evidence from randomized controlled trials involving collegiate and university athletes supports these mechanisms. Li (54) and Li (55) reported that *G. lucidum* polysaccharide oral liquid (10 mL twice daily, 6 days on/1 day off for 90 days) significantly increased hemoglobin and hematocrit levels, improved total work output, and reduced blood lactate and blood urea nitrogen (BUN), while enhancing antioxidant enzyme activities (SOD, CAT) and immunoglobulin levels (IgG, IgA, IgM). Tang (56) and Zhao (57) observed that triterpenoid or polysaccharide capsules (75–750 mg/day for 30 days) effectively elevated red blood cell count, hemoglobin, and SOD activity, while decreasing heart rate, lactate, BUN, AST, LDH, and lipid peroxide (LPO) concentrations, indicating improved antioxidant defense and accelerated recovery. Liu (58) further demonstrated that daily supplementation with Ganoderma extract (150 mg/day for 8 weeks) significantly increased grip strength and VO₂max without adverse biochemical changes, suggesting enhanced cardiopulmonary fitness and a good safety profile.

Collectively, these studies indicate that *G. lucidum* supplementation can improve aerobic capacity, reduce fatigue-related metabolites, and strengthen immune and antioxidant defenses in athletes. The most frequently reported biochemical improvements include increased hemoglobin and SOD activity and decreased lactate and BUN levels. A meta-analysis based on these trials revealed consistent benefits across these parameters, suggesting that *G. lucidum* polysaccharides and triterpenoids may serve as effective ergogenic and recovery-enhancing agents for trained individuals.

#### 4.1.3 Oyster mushrooms

Oyster mushrooms are among the most popular edible mushrooms worldwide and rank third in globally cultivated mushroom production (59). Various bioactive com-pounds extracted from oyster mushrooms, such as polysaccharides, peptides, glycoproteins, phenolic compounds, and lipids, have been shown to exhibit a wide range of bio-logical activities, including antioxidant, anti-inflammatory, analgesic, antitumor, and antihypertensive effects (60–63). Two studies have demonstrated that *β*-glucan derived from *P. ostreatus*, administered at a daily dose of 100 mg, exerts beneficial immunomodulatory effects in high-intensity physical training athletes (Figure 5C). One study showed that 2 months of supplementation prevented the post-exercise decline in NK cell activity (24), whereas an-other found that 3 months of *P. ostreatus* intake significantly reduced the incidence of up-per respiratory tract infection symptoms and maintained phagocytic function (64).

β-glucan from mushrooms is recognized by immune receptors such as Dectin-1 and Complement Receptor 3 (CR3) on macrophages, neutrophils, and NK cells (65). Upon binding, these receptors initiate a MyD88-dependent signaling cascade (often involving Toll-like receptors as co-receptors) that leads to activation of NF-κB and MAPK pathways in immune cells (66). The result is an increased release of cytokines like IL-6, IL-12, and IFN-*γ*, which in turn enhances the cytotoxicity of NK cells and the microbicidal activity of phagocytes (65). Moreover, research shows that soluble β-glucans binding to CR3 on NK cells and neutrophils improve their ability to engulf and destroy targets. Oyster mushroom polysaccharides also have antioxidative effects, scavenging free radicals and reducing exercise-induced inflammation. Overall, *Pleurotus ostreatus* supplementation can be seen as a natural “immune insurance” for athletes. Future work may explore optimal dosing and timing to maximize oyster mushrooms’ benefits in athletic populations contribute to improved immunity or metabolism during training.

#### 4.1.4 Cordyceps militaris

*Cordyceps militaris*, which is similar to *C. sinensis*, is a unique and valuable medicinal fungus. It shares similar chemical constituents and pharmacological properties with *C. sinensis* and is a cost-effective alternative to the more expensive natural *C. sinensis* (67, 68). *C. militaris* also contains cordycepin, which exhibits antioxidant activity and modulates various molecular targets, including transcription factors, matrix metalloproteinases, components of the complement cascade, and adhesion molecules such as intercellular adhesion molecule-1, selectins, and vascular cell adhesion molecule-1. These interactions represent the mechanisms underlying its anti-inflammatory effects (37, 69, 70). These biological activities may explain the physiological effects observed in athletes. Notably, a recent placebo-controlled trial demonstrated that supplementation with *C. militaris* mycelium extract over a 16-week pre-season training period helped preserve hemoglobin and hematocrit levels and reduced markers of muscle damage in long-distance runners (71). Cordyceps prevented the depletion of iron stores and thus protected hemoglobin levels. This aligns with Cordyceps’ known anti-inflammatory action: by reducing IL-6 and related inflammatory signals, it likely blunted the upregulation of hepcidin (the iron-regulatory hormone), thereby allowing normal iron availability for erythropoiesis. Indeed, an 8-week study in healthy adults found Cordyceps intake lowered pro-inflammatory cytokines (72). Cordycepin attenuated the decline in serum ferritin levels and lowered elevated creatine kinase levels, suggesting its potential role in supporting iron metabolism and protecting against exercise-induced muscle injury. In cell studies, cordycepin blocks TNF-*α* induced NF-κB activation by preventing the phosphorylation and degradation of IκBα (the inhibitor of NF-κB) and by impeding the ubiquitination of the NF-κB complex (73). This leads to reduced expression of inflammatory genes, including those coding for adhesion molecules (ICAM-1, VCAM-1) and enzymes like iNOS. By downregulating NF-κB and p38 MAPK pathways, cordycepin curtails the cascade that normally would result in leukocyte adhesion and muscle inflammatory damage during intense exercise (74). It also modulates other molecular targets: studies report cordycepin can decrease levels of matrixmetalloproteinases and components of the complement system that contribute to inflammation-induced tissue injury (69).

#### 4.1.5 Tremella fuciformis

*Tremella fuciformis*, commonly known as snow fungus, has become one of the most widely cultivated edible mushrooms in China (53). It is characterized by low energy and fat content but is rich in proteins, polysaccharides, and dietary fiber, along with a variety of minerals, trace elements, and vitamins (75). In many Asian countries, *T. fuciformis* is valued both as a functional food and a traditional herbal ingredient, often consumed as a tonic. Recent studies have identified *T. fuciformis* polysaccharides (TPS) as the principal bioactive components responsible for its nutritional and health-promoting properties. These polysaccharides exhibit a broad range of biological activities, including immunomodulatory, hypoglycemic, hypolipidemic, anti-aging, anti-ulcer, antithrombotic, and antimutagenic effects (75).

In a controlled study of university basketball players, Wang (76) found that 3 weeks of TPS supplementation increased serum immunoglobulin levels (IgA, IgG, and IgM) and decreased the inflammatory cytokines TNF-*α* and IL-10. Fasting blood glucose was also reduced, with the most pronounced changes observed in the high-dose group. These results suggest that TPS can help counteract exercise-induced immune suppression and improve energy metabolism during intensive training. Mechanistically, TPS may enhance immune defense by activating macrophages and promoting cytokine secretion through the TLR4–NF-κB signaling pathway (77, 78). TPS may strengthen immune resilience, reduce inflammation, and support better metabolic adaptation in athletes engaged in high-intensity exercise.

TPS, like many mushroom polysaccharides, cannot directly enter cells but instead interacts with immune cells via surface receptors (79). Specifically, Tremella polysaccharides activate macrophages by engaging Toll-like receptor 4 (TLR4) on the cell surface, which triggers the NF-κB signaling pathway inside the cells (77). Activated NF-κB leads to increased production of cytokinesthat orchestrate a more robust immune response. Interestingly, studies have found that certain chemical modifications of TPS, like acetylation, can enhance this TLR4/NF-κB activation and thereby amplify cytokine secretion (77). This explains how TPS might boost immunoglobulin levels: macrophage and dendritic-cell activation provides the necessary signals (e.g., IL-6, BAFF) for B cells to expand and secrete antibodies. Moreover, TPS appears to modulate immune balance by affecting regulatory pathways. One study showed that TPS treatment inhibited the expression of microRNA-155 in LPS-stimulated macrophages, which in turn prevented over-activation of NF-κB (80). miR-155 is a pro-inflammatory microRNA, and its suppression by TPS resulted in reduced inflammatory mediator release (78). Through this miRNA-mediated mechanism, Tremella exhibits an anti-inflammatory effect, ensuring that immune activation does not overshoot into chronic inflammation. Beyond immunity, Tremella polysaccharides have notable effects on metabolism. Their hypoglycemic activity is partly due to their soluble fiber nature, as TPS increases the viscosity of intestinal contents and can inhibit α-amylase, slowing carbohydrate digestion and absorption (81).

#### 4.1.6 Mechanistic insights

The beneficial effects of fungal supplements in athletes appear to arise from three interrelated mechanisms: antioxidant defense, immune regulation, and metabolic adaptation. Polysaccharides, triterpenoids, and cordycepin enhance endogenous antioxidant capacity by increasing superoxide dismutase, catalase, and glutathione peroxidase activities, thereby reducing exercise-induced oxidative stress and inflammation through the inhibition of NF-κB and MAPK pathways. *β*-glucans and other polysaccharides interact with immune receptors such as Dectin-1, CR3, and TLR4, activating MyD88-dependent signaling cascades that enhance macrophage activity, cytokine secretion, and immunoglobulin production, which together stabilize immune homeostasis under intense training. Furthermore, cordycepin promotes mitochondrial oxidative phosphorylation and erythropoiesis by stimulating cAMP/PKA-mediated steroidogenesis, while Ganoderma polysaccharides improve hemoglobin levels, oxygen transport, and recovery efficiency. Collectively, these molecular mechanisms suggest that fungal supplementation enhances physiological resilience by coordinating antioxidant, immune, and metabolic responses, contributing to improved endurance, faster recovery, and reduced fatigue in athletes.

### 4.2 Adverse effects of fungal supplements

Human trials indicate that mushroom-based supplements are generally well tolerated, with very few reported side effects. In the studies included in this review, only three trials mentioned adverse events, and those reports were qualitative rather than systematic. Common mild symptoms, such as gastrointestinal discomfort or insomnia, were not consistently recorded across trials, which limits the ability to fully evaluate the balance between risks and benefits. Bergendiova et al. (64) observed no side effects with insoluble β-glucan derived from *Pleurotus ostreatus* in athletes. Similarly, Zhang et al. (49) and Thongsawang et al. (23) reported no evidence of hepatotoxicity after *Cordyceps sinensis* supplementation. These findings align with the long history of *Ganoderma lucidum*, *P. ostreatus*, *C. sinensis*, and *C. militaris* being used as food and in traditional medicine, suggesting that short-term consumption of these edible fungi is unlikely to cause significant harm.

However, broader clinical evidence shows that mild adverse reactions may occur in some populations. A Cochrane systematic review on *G. lucidum* in cancer patients reported minor gastrointestinal symptoms, such as nausea, constipation, and sleep disturbance, although no serious hepatic or renal toxicity was observed (82). While these events are infrequent and transient, rare cases of liver injury associated with Reishi extract have been reported, emphasizing that “natural” does not always guarantee absolute safety (83).

Despite the generally benign safety profile observed, a critical limitation in the current literature is the insufficient monitoring and reporting of adverse events in many sports nutrition studies. Only a few of the trials included in this review systematically collected safety data, and most failed to specify whether adverse events were monitored throughout the intervention period. This pattern is consistent with broader observations in sports supplementation research, where harms are often underreported or omitted from publication. For example, Kreider et al. (84) analyzed more than 200 creatine supplementation trials and found that only 13.7% mentioned any side effects in intervention groups, while approximately 86% of trials reported no adverse events at all. A meta-epidemiological review by Zeijlon et al. (85) also demonstrated that reports of supplement-related adverse events frequently lacked essential details, making it difficult to determine causality or severity. This underreporting trend may be driven by the tendency of investigators to focus on ergogenic effects while assuming the safety of nutritional supplements.

The lack of standardized adverse event reporting has several implications for research involving athletes. Even mild symptoms, such as gastrointestinal upset, headache, or disturbed sleep, can influence training quality, recovery dynamics, and adherence to supplementation. Without systematic monitoring, these subtle yet relevant effects may remain undetected. Furthermore, the intervention durations in current trials are relatively short, typically lasting no longer than 12 to 16 weeks, which prevents the evaluation of long-term safety, cumulative toxicity, or possible nutrient interactions. Athletes often consume supplements over extended seasons or training cycles, highlighting the need for longitudinal monitoring of adverse outcomes. In summary, existing evidence suggests that edible fungal supplements are well tolerated in the short term, with minimal and non-serious adverse events reported.

### 4.3 Strengths of the study

This review had several strengths. The protocol was prospectively registered, and a rigorous systematic search was conducted across three major scientific databases: PubMed, Scopus, and Web of Science, as well as the Chinese database China National Knowledge Infrastructure (CNKI). Only high-quality randomized controlled trials were included, and selection was restricted to studies that examined fungal supplements as the sole intervention, excluding those involving combined supplementation. Although the included studies spanned from 2004 to 2024, this time range reflects the limited number of high-quality randomized controlled trials available in this field. The inclusion of earlier studies ensured a more comprehensive understanding of the long-term development and consistency of findings regarding fungal supplementation in athletes.

### 4.4 Limitations and future directions

The heterogeneity of supplement characteristics limited the comparability of outcomes. Considerable variation existed among the included trials in terms of extraction methods, purity, and concentrations of active compounds. The lack of detailed reporting on *β*-glucan extraction protocols and the molecular-weight distribution of *G. lucidum* polysaccharides has hindered cross-study interpretation. Establishing standardized characterization and reporting frameworks for fungal supplements is therefore essential to improve reproducibility and comparability across studies.

The number of eligible randomized controlled trials was limited after full-text screening, primarily due to strict inclusion criteria focusing on single-fungus interventions under controlled designs. This ensured methodological rigor but inevitably constrained the overall evidence base. Future research should broaden the scope and scale of investigation by conducting larger, well-controlled trials that adopt standardized methodologies to validate the potential ergogenic, immunomodulatory, and hematological benefits of fungal supplementation in athletes. Finally, the predominance of positive findings raises the likelihood of publication bias. Among the 14 included trials, 13 reported favorable outcomes, suggesting that the true effect size may be overestimated. Future meta-analyses should incorporate gray literature and unpublished data and encourage the transparent reporting of neutral or negative results to achieve a more balanced and reliable understanding of the efficacy of fungal supplementation.

### 4.5 Guidelines and recommendations

The findings of the present meta-analysis, together with the synthesis of randomized controlled trials, provide several practical recommendations regarding the use of fungal supplements among athletes. The pooled quantitative evidence indicated that *C. sinensis* supplementation at a dosage of 2 to 3 grams per day for 6 to 12 weeks significantly improved endurance performance, ventilatory threshold, and VO_2_peak, demonstrating enhanced aerobic capacity and oxygen utilization efficiency. These effects were accompanied by low heterogeneity, suggesting stable and reproducible benefits across studies. Therefore, *C. sinensis* may be particularly suitable for endurance athletes such as runners and marathoners during pre-season or base training phases to strengthen aerobic metabolism and delay fatigue. When using *C. sinensis*, the cultivation origin and cordycepin content should be verified, as these factors are directly related to its physiological efficacy.

The subgroup meta-analysis further revealed that *G. lucidum* supplementation, within a dosage range of 75 mg to 5 g per day, significantly increased hemoglobin concentration and hematocrit, while reducing blood lactate and blood urea nitrogen levels and elevating superoxide dismutase activity. These results indicate that *G. lucidum* can improve erythropoietic function, post-exercise recovery, and antioxidant capacity. The supplementation appears particularly beneficial for athletes undergoing intensive training or hypoxic exposure, such as endurance cyclists or football players. Based on the available evidence, a continuous intake of standardized *G. lucidum* extract (150 mg to 5 g daily for 8–12 weeks) may contribute to improved oxygen transport, reduced fatigue, and enhanced recovery.

From a practical standpoint, athletes are encouraged to select fungal supplements produced under standardized extraction and quality-control conditions. The quantification of key bioactive components, including polysaccharides, triterpenoids, *β*-glucans, and cordycepin, should be clearly specified by the manufacturer. Overall, the results of this meta-analysis support the ergogenic, antioxidant, and immunoprotective potential of selected fungal supplements when used within evidence-based dosage and duration ranges. Nevertheless, large-scale randomized controlled trials with standardized formulations and extended follow-up are still needed to refine these practical recommendations and establish formal clinical guidelines for sports nutrition.

## 5 Conclusion

This review, which included 14 studies, indicates that fungal supplements contain various bioactive compounds such as polysaccharides, cordycepin, and *β*-glucans, which are believed to exert antioxidant, anti-inflammatory, immunomodulatory, and hematological effects in athletes. These compounds are associated with beneficial outcomes, including enhanced endurance, improved immune function, and reduced muscle damage markers. Meta-analysis results confirmed significant improvements in aerobic capacity and erythropoiesis, particularly with supplementation of *Cordyceps sinensis* and *Ganoderma lucidum*. Moreover, current evidence suggests good safety profiles, with no serious adverse effects reported. Future studies should employ standardized formulations, longer intervention durations, and omics-based approaches to elucidate the molecular mechanisms and dose response relationships of fungal bioactive compounds across different athletic populations.

## References

[1] Xu J. Assessing global fungal threats to humans. mLife. (2022) 1:223–40. doi: 10.1002/mlf2.12036, PMID: 38818220 PMC10989982

[2] Coleine C Stajich JE Selbmann L. Fungi are key players in extreme ecosystems. Trends Ecol Evol. (2022) 37:517–28. doi: 10.1016/j.tree.2022.02.002, PMID: 35246323

[3] Adeyeye SAO. Fungal mycotoxins in foods: a review. Cogent Food Agric. (2016) 2:3127. doi: 10.1080/23311932.2016.1213127

[4] El-Ramady H Abdalla N Badgar K Llanaj X Törős G Hajdú P . Edible mushrooms for sustainable and healthy human food: nutritional and medicinal attributes. Sustainability. (2022) 14:4941. doi: 10.3390/su14094941

[5] Zhang Y Wang D Chen Y Liu T Zhang S Fan H . Healthy function and high valued utilization of edible fungi. Food Sci Human Wellness. (2021) 10:408–20. doi: 10.1016/j.fshw.2021.04.003

[6] Chen H-Y Lei J-Y Li S-L Guo L-Q Lin J-F Wu G-H . Progress in biological activities and biosynthesis of edible fungi terpenoids. Crit Rev Food Sci Nutr. (2023) 63:7288–310. doi: 10.1080/10408398.2022.2045559, PMID: 35238261

[7] Kumar K Mehra R Guiné RPF Lima MJ Kumar N Kaushik R . Edible mushrooms: a comprehensive review on bioactive compounds with health benefits and processing aspects. Foods. (2021) 10:2996. doi: 10.3390/foods10122996, PMID: 34945547 PMC8700757

[8] Arslan NP Dawar P Albayrak S Doymus M Azad F Esim N . Fungi-derived natural antioxidants. Crit Rev Food Sci Nutr. (2025) 65:1593–616. doi: 10.1080/10408398.2023.2298770, PMID: 38156661

[9] Yu W Zhang Y Yao L Peng J Tu Y He B. Research progress on the prevention of tumor by fungal polysaccharides. Trends Food Sci Technol. (2024) 147:104422. doi: 10.1016/j.tifs.2024.104422

[10] Ma X Guo DD Peterson EC Dun Y Li DY. Structural characterization and anti-aging activity of a novel extracellular polysaccharide from fungus Phellinus sp. in a mammalian system. Food Funct. (2016) 7:3468–79. doi: 10.1039/C6FO00422A, PMID: 27405813

[11] Schneider I Kressel G Meyer A Krings U Berger RG Hahn A. Lipid lowering effects of oyster mushroom (*Pleurotus ostreatus*) in humans. J Funct Foods. (2011) 3:17–24. doi: 10.1016/j.jff.2010.11.004

[12] Aramabašić Jovanović J Mihailović M Uskoković A Grdović N Dinić S Vidaković M. The effects of major mushroom bioactive compounds on mechanisms that control blood glucose level. J Fungi. (2021) 7:58. doi: 10.3390/jof7010058, PMID: 33467194 PMC7830770

[13] Guillamón E García-Lafuente A Lozano M D’Arrigo M Rostagno MA Villares A . Edible mushrooms: role in the prevention of cardiovascular diseases. Fitoterapia. (2010) 81:715–23. doi: 10.1016/j.fitote.2010.06.005 20550954

[14] Luti S Modesti A Modesti PA. Inflammation, peripheral signals and redox homeostasis in athletes who practice different sports. Antioxidants. (2020) 9:1065. doi: 10.3390/antiox9111065, PMID: 33143147 PMC7693221

[15] Magherini F Fiaschi T Marzocchini R Mannelli M Gamberi T Modesti PA . Oxidative stress in exercise training: the involvement of inflammation and peripheral signals. Free Radic Res. (2019) 53:1155–65. doi: 10.1080/10715762.2019.1697438, PMID: 31762356

[16] Slattery K Bentley D Coutts AJ. The role of oxidative, inflammatory and neuroendocrinological systems during exercise stress in athletes: implications of antioxidant supplementation on physiological adaptation during intensified physical training. Sports Med. (2015) 45:453–71. doi: 10.1007/s40279-014-0282-7, PMID: 25398224

[17] Sabzevari Rad R. The impact of different training intensities on athletes’ immune system function and the management of upper respiratory traction infections: a narrative review. Sport Sci Health. (2024) 20:415–26. doi: 10.1007/s11332-023-01110-7

[18] Perez-Montilla J Cuevas-Cervera M Gonzalez-Muñoz A Garcia-Rios M Navarro-Ledesma S. Efficacy of nutritional strategies on the improvement of the performance and health of the athlete: a systematic review. IJERPH. (2022) 19:4240. doi: 10.3390/ijerph19074240, PMID: 35409921 PMC8998415

[19] Shu M-Y Liang J Jo Y-J Eom S-H Kim C-H. Applications and benefits of dietary supplements in taekwondo: a systematic review. Life. (2025) 15:559. doi: 10.3390/life15040559, PMID: 40283114 PMC12028964

[20] Parnian-Khajehdizaj N Mainer Pardos E Machek SB Nobari H. Association between *Pleurotus ostreatus* consumption and more optimal sports performance: a narrative review. J Food Biochem. (2024) 2024:2610415. doi: 10.1155/2024/2610415

[21] Page MJ McKenzie JE Bossuyt PM Boutron I Hoffmann TC Mulrow CD . The PRISMA 2020 statement: an updated guideline for reporting systematic reviews. BMJ. (2021) 372:n71. doi: 10.1136/bmj.n71, PMID: 33782057 PMC8005924

[22] Minozzi S Cinquini M Gianola S Gonzalez-Lorenzo M Banzi R. The revised Cochrane risk of bias tool for randomized trials (RoB 2) showed low interrater reliability and challenges in its application. J Clin Epidemiol. (2020) 126:37–44. doi: 10.1016/j.jclinepi.2020.06.015, PMID: 32562833

[23] Thongsawang S Krataithong T ChorCharoenying S Norchai P Nokkaew N. Applying *Cordyceps sinensis* to boost endurance performance in long-distance runners. J Exerc Physiol Online. (2021) 24:1–13.

[24] Bobovčák M Kuniaková R Gabriž J Majtán J. Effect of Pleuran (β-glucan from *Pleurotus ostreatus*) supplementation on cellular immune response after intensive exercise in elite athletes. Appl Physiol Nutr Metab. (2010) 35:755–62. doi: 10.1139/H10-070, PMID: 21164546

[25] Savioli FP Zogaib P Franco E Alves De Salles FC Giorelli GV Andreoli CV. Effects of *Cordyceps sinensis* supplementation during 12 weeks in amateur marathoners: a randomized, double-blind placebo-controlled trial. J Herbal Med. (2022) 34:100570. doi: 10.1016/j.hermed.2022.100570

[26] Liu Y Wang J Wang W Zhang H Zhang X Han C. The chemical constituents and pharmacological actions of *Cordyceps sinensis*. Evid Based Complement Alternat Med. (2015) 2015:1–12. doi: 10.1155/2015/575063, PMID: 25960753 PMC4415478

[27] Yang FQ Guan J Li SP. Fast simultaneous determination of 14 nucleosides and nucleobases in cultured cordyceps using ultra-performance liquid chromatography. Talanta. (2007) 73:269–73. doi: 10.1016/j.talanta.2007.03.034, PMID: 19073027

[28] Zhou X Luo L Dressel W Shadier G Krumbiegel D Schmidtke P . Cordycepin is an immunoregulatory active ingredient of *Cordyceps sinensis*. Am J Chin Med. (2008) 36:967–80. doi: 10.1142/S0192415X08006387, PMID: 19051361

[29] Du X-F Song Z-W. Effect of *Cordyceps sinensis* polysaccharide extract on the complement system of female wrestlers. Edible Fungi China. (2019) 38:27–9. doi: 10.13629/j.cnki.53-1054.2019.02.008

[30] Leu S-F Poon SL Pao H-Y Huang B-M. The *in vivo* and *in vitro* stimulatory effects of cordycepin on mouse Leydig cell steroidogenesis. Biosci Biotechnol Biochem. (2011) 75:723–31. doi: 10.1271/bbb.100853, PMID: 21512251

[31] Coates AM Joyner MJ Little JP Jones AM Gibala MJ. A perspective on high-intensity interval training for performance and health. Sports Med. (2023) 53:85–96. doi: 10.1007/s40279-023-01938-6, PMID: 37804419 PMC10721680

[32] Alghannam AF Ghaith MM Alhussain MH. Regulation of energy substrate metabolism in endurance exercise. IJERPH. (2021) 18:4963. doi: 10.3390/ijerph18094963, PMID: 34066984 PMC8124511

[33] Coyle EF. Physiological determinants of endurance exercise performance. J Sci Med Sport. (1999) 2:181–9. doi: 10.1016/S1440-2440(99)80172-8, PMID: 10668757

[34] Parcell AC Smith JM Schulthies SS Myrer JW Fellingham G. *Cordyceps sinensis* (CordyMax cs-4) supplementation does not improve endurance exercise performance. Int J Sport Nutr Exerc Metab. (2004) 14:236–42. doi: 10.1123/ijsnem.14.2.236, PMID: 15118196

[35] Yang FQ Li DQ Feng K Hu DJ Li SP. Determination of nucleotides, nucleosides and their transformation products in cordyceps by ion-pairing reversed-phase liquid chromatography–mass spectrometry. J Chromatogr A. (2010) 1217:5501–10. doi: 10.1016/j.chroma.2010.06.062, PMID: 20637470

[36] Zhang L Zhou S Bi B Wang H Fu B Guo M . Cordycepin suppresses steroidogenic acute regulatory protein expression by reducing SP1 in human granulosa-lutein cells. Reproduction. (2024) 168:e240240. doi: 10.1530/REP-24-0240, PMID: 39133156

[37] Qin P Li X Yang H Wang Z-Y Lu D. Therapeutic potential and biological applications of cordycepin and metabolic mechanisms in cordycepin-producing fungi. Molecules. (2019) 24:2231. doi: 10.3390/molecules24122231, PMID: 31207985 PMC6632035

[38] Tian H-Y Yu D-J Xie T Xu M-X Wang Y-H Sun X-L . Cordycepin alleviates metabolic dysfunction-associated liver disease by restoring mitochondrial homeostasis and reducing oxidative stress via parkin-mediated mitophagy. Biochem Pharmacol. (2025) 232:116750. doi: 10.1016/j.bcp.2025.116750, PMID: 39793718

[39] Millet GP Vleck VE Bentley DJ. Physiological differences between cycling and running: lessons from triathletes. Sports Med. (2009) 39:179–206. doi: 10.2165/00007256-200939030-00002, PMID: 19290675

[40] Shashidhar MG Giridhar P Udaya Sankar K Manohar B. Bioactive principles from *Cordyceps sinensis*: a potent food supplement – a review. J Funct Foods. (2013) 5:1013–30. doi: 10.1016/j.jff.2013.04.018, PMID: 32288795 PMC7104994

[41] Peeling P Binnie MJ Goods PSR Sim M Burke LM. Evidence-based supplements for the enhancement of athletic performance. Int J Sport Nutr Exerc Metab. (2018) 28:178–87. doi: 10.1123/ijsnem.2017-0343, PMID: 29465269

[42] Bishop KS Kao CHJ Xu Y Glucina MP Paterson RRM Ferguson LR. From 2000years of *Ganoderma lucidum* to recent developments in nutraceuticals. Phytochemistry. (2015) 114:56–65. doi: 10.1016/j.phytochem.2015.02.015, PMID: 25794896

[43] Sharma C Bhardwaj N Sharma A Tuli HS Batra P Beniwal V . Bioactive metabolites of *Ganoderma lucidum*: factors, mechanism and broad spectrum therapeutic potential. J Herbal Med. (2019) 17-18:100268. doi: 10.1016/j.hermed.2019.100268, PMID: 41146674

[44] Swallah MS Bondzie-Quaye P Wu Y Acheampong A Sossah FL Elsherbiny SM . Therapeutic potential and nutritional significance of *Ganoderma lucidum* – a comprehensive review from 2010 to 2022. Food Funct. (2023) 14:1812–38. doi: 10.1039/D2FO01683D, PMID: 36734035

[45] Ahmad MF Alsayegh AA Ahmad FA Akhtar MS Alavudeen SS Bantun F . *Ganoderma lucidum*: insight into antimicrobial and antioxidant properties with development of secondary metabolites. Heliyon. (2024) 10:e25607. doi: 10.1016/j.heliyon.2024.e25607, PMID: 38356540 PMC10865332

[46] Gao X Homayoonfal M. Exploring the anti-cancer potential of *Ganoderma lucidum* polysaccharides (GLPs) and their versatile role in enhancing drug delivery systems: a multifaceted approach to combat cancer. Cancer Cell Int. (2023) 23:324. doi: 10.1186/s12935-023-03146-8, PMID: 38104078 PMC10724890

[47] Seweryn E Ziała A Gamian A. Health-promoting of polysaccharides extracted from *Ganoderma lucidum*. Nutrients. (2021) 13:2725. doi: 10.3390/nu13082725, PMID: 34444885 PMC8400705

[48] Brocherie F Girard O Faiss R Millet GP. High-intensity intermittent training in hypoxia: a double-blinded, placebo-controlled field study in youth football players. J Strength Cond Res. (2015) 29:226–37. doi: 10.1519/JSC.0000000000000590, PMID: 24978836

[49] Zhang Y Lin Z Hu Y Wang F. Effect of *Ganoderma lucidum* capsules on T lymphocyte subsets in football players on “living high−training low.”. Br J Sports Med. (2008) 42:819–22. doi: 10.1136/bjsm.2007.038620, PMID: 18048435

[50] Lu J He R Sun P Zhang F Linhardt RJ Zhang A. Molecular mechanisms of bioactive polysaccharides from *Ganoderma lucidum* (Lingzhi), a review. Int J Biol Macromol. (2020) 150:765–74. doi: 10.1016/j.ijbiomac.2020.02.035, PMID: 32035956

[51] Xu J Shen R Jiao Z Chen W Peng D Wang L . Current advancements in antitumor properties and mechanisms of medicinal components in edible mushrooms. Nutrients. (2022) 14:2622. doi: 10.3390/nu14132622, PMID: 35807802 PMC9268676

[52] Shi M Yang Y Hu X Zhang Z. Effect of ultrasonic extraction conditions on antioxidative and immunomodulatory activities of a *Ganoderma lucidum* polysaccharide originated from fermented soybean curd residue. Food Chem. (2014) 155:50–6. doi: 10.1016/j.foodchem.2014.01.037, PMID: 24594153

[53] Zhang J Gao X Pan Y Xu N Jia L. Toxicology and immunology of *Ganoderma lucidum* polysaccharides in Kunming mice and Wistar rats. Int J Biol Macromol. (2016) 85:302–10. doi: 10.1016/j.ijbiomac.2015.12.090, PMID: 26763176

[54] Li J-S. The influence of Ganoderma polysaccharides on training effect and immune function athletes. J Inner Mongolia Normal Univ. (2016) 45:72–5.

[55] Li X-Y. Effect of *Ganoderma lucidum* polysaccharide on exercise fatigue and exercise-induced immunosuppression. Edible Fungi China. (2020) 39:45–8. doi: 10.13629/j.cnki.53-1054.2020.02.013

[56] Tang C-Q. Effect of *Ganoderma lucidum* triterpenes on recovery of athletes’physical fitness. Edible Fungi China. (2020) 39:207–10. doi: 10.13629/j.cnki.53-1054.2020.07.060

[57] Zhao L. Effect of *Ganoderma lucidum* polysaccharides on athletes’anti-lipid peroxidation. Edible Fungi China. (2021) 40:75–9. doi: 10.13629/j.cnki.53-1054.2021.02.014

[58] Liu Y. The effect of *Ganoderma* spp. extract on physical fitness of athletes. Edible Fungi China. (2020) 39:232–4. doi: 10.13629/j.cnki.53-1054.2020.04.071

[59] Corrêa RCG Brugnari T Bracht A Peralta RM Ferreira ICFR. Biotechnological, nutritional and therapeutic uses of *Pleurotus* spp. (oyster mushroom) related with its chemical composition: a review on the past decade findings. Trends Food Sci Technol. (2016) 50:103–17. doi: 10.1016/j.tifs.2016.01.012

[60] Abidin MHZ Abdullah N Abidin NZ. Therapeutic properties of *Pleurotus* species (oyster mushrooms) for atherosclerosis: a review. Int J Food Prop. (2017) 20:1251–61. doi: 10.1080/10942912.2016.1210162

[61] Devi PV Islam J Narzary P Sharma D Sultana F. Bioactive compounds, nutraceutical values and its application in food product development of oyster mushroom. J Future Foods. (2024) 4:335–42. doi: 10.1016/j.jfutfo.2023.11.005

[62] Greeshma P Ravikumar KS Neethu MN Pandey M Zuhara KF Janardhanan KK. Antioxidant, anti-inflammatory, and antitumor activities of cultured mycelia and fruiting bodies of the elm oyster mushroom, Hypsizygus ulmarius (agaricomycetes). Int J Med Mushrooms. (2016) 18:235–44. doi: 10.1615/IntJMedMushrooms.v18.i3.60, PMID: 27481157

[63] Ray P Kundu S Paul D. Exploring the therapeutic properties of Chinese mushrooms with a focus on their anti-cancer effects: a systemic review. Pharmacol Res Mod Chin Med. (2024) 11:100433. doi: 10.1016/j.prmcm.2024.100433

[64] Bergendiova K Tibenska E Majtan J. Pleuran (β-glucan from Pleurotus ostreatus) supplementation, cellular immune response and respiratory tract infections in athletes. Eur J Appl Physiol. (2011) 111:2033–40. doi: 10.1007/s00421-011-1837-z, PMID: 21249381

[65] Noorbakhsh Varnosfaderani SM Ebrahimzadeh F Akbari Oryani M Khalili S Almasi F Mosaddeghi Heris R . Potential promising anticancer applications of β-glucans: a review. Biosci Rep. (2024) 44:BSR20231686. doi: 10.1042/BSR20231686, PMID: 38088444 PMC10776902

[66] Zhao L Geng Z Wang Y Wen J Liu D. Immunomodulatory effects of *Ganoderma lucidum* bioactive compounds on gut–brain and gut–liver axis disorders. Curr Issues Mol Biol. (2025) 47:842. doi: 10.3390/cimb47100842, PMID: 41150791 PMC12563824

[67] Krishna KV Balasubramanian B Park S Bhattacharya S Kadanthottu Sebastian J Liu W-C . Conservation of endangered *Cordyceps sinensis* through artificial cultivation strategies of *C. militaris*, an alternate. Mol Biotechnol. (2025) 67:1382–97. doi: 10.1007/s12033-024-01154-1, PMID: 38658470

[68] Zhang J Wen C Duan Y Zhang H Ma H. Advance in cordyceps militaris (Linn) link polysaccharides: isolation, structure, and bioactivities: a review. Int J Biol Macromol. (2019) 132:906–14. doi: 10.1016/j.ijbiomac.2019.04.020, PMID: 30954592

[69] Shweta Abdullah S Komal Kumar A. A brief review on the medicinal uses of *Cordyceps militaris*. Pharmacol Res Mod Chin Med. (2023) 7:100228. doi: 10.1016/j.prmcm.2023.100228

[70] Tan L Song X Ren Y Wang M Guo C Guo D . Anti-inflammatory effects of cordycepin: a review. Phytother Res. (2021) 35:1284–97. doi: 10.1002/ptr.6890, PMID: 33090621

[71] Nakamura A Shinozaki E Suzuki Y Santa K Kumazawa Y Kobayashi F . Effect of the Administration of Cordyceps militaris mycelium extract on blood markers for Anemia in long-distance runners. Nutrients. (2024) 16:1835. doi: 10.3390/nu16121835, PMID: 38931190 PMC11206946

[72] Ontawong A Pengnet S Thim-Uam A Munkong N Narkprasom N Narkprasom K . A randomized controlled clinical trial examining the effects of cordyceps militaris beverage on the immune response in healthy adults. Sci Rep. (2024) 14:7994. doi: 10.1038/s41598-024-58742-z, PMID: 38580687 PMC10997757

[73] Ren Z Cui J Huo Z Xue J Cui H Luo B . Cordycepin suppresses TNF-α-induced NF-κB activation by reducing p65 transcriptional activity, inhibiting IκBα phosphorylation, and blocking IKKγ ubiquitination. Int Immunopharmacol. (2012) 14:698–703. doi: 10.1016/j.intimp.2012.10.008, PMID: 23102662

[74] Yan L-J Yang H-T Duan H-Y Wu J-T Qian P Fan X-W . Cordycepin inhibits vascular adhesion molecule expression in TNF-α-stimulated vascular muscle cells. Exp Ther Med. (2017) 14:2335–40. doi: 10.3892/etm.2017.4746, PMID: 28962164 PMC5609172

[75] Wu Y Wei Z Zhang F Linhardt RJ Sun P Zhang A. Structure, bioactivities and applications of the polysaccharides from *Tremella fuciformis* mushroom: a review. Int J Biol Macromol. (2019) 121:1005–10. doi: 10.1016/j.ijbiomac.2018.10.117, PMID: 30342120

[76] Wang D-L. Regulatory effect of *Tremella fuciformis* polysaccharides on basketball players’ exercise-induced immunosuppression. Edible Fungi China. (2020) 39:199–203. doi: 10.13629/j.cnki.53-1054.2020.08.056

[77] Huang T-Y Yang F-L Chiu H-W Chao H-C Yang Y-J Sheu J-H . An immunological polysaccharide from *Tremella fuciformis*: essential role of acetylation in immunomodulation. IJMS. (2022) 23:10392. doi: 10.3390/ijms231810392, PMID: 36142298 PMC9499394

[78] Ruan Y Li H Pu L Shen T Jin Z. *Tremella fuciformis* polysaccharides attenuate oxidative stress and inflammation in macrophages through miR-155. Anal Cell Pathol. (2018) 2018:1–10. doi: 10.1155/2018/5762371, PMID: 29854576 PMC5954968

[79] He G Chen T Huang L Zhang Y Feng Y Qu S . *Tremella fuciformis* polysaccharide reduces obesity in high-fat diet-fed mice by modulation of gut microbiota. Front Microbiol. (2022) 13:1073350. doi: 10.3389/fmicb.2022.1073350, PMID: 36545204 PMC9760882

[80] Yin Z Zhang J Qin J Guo L Guo Q Kang W . Anti-inflammatory properties of polysaccharides from edible fungi on health-promotion: a review. Front Pharmacol. (2024) 15:1447677. doi: 10.3389/fphar.2024.1447677, PMID: 39130633 PMC11310034

[81] Zhang X Fu Y Wang H Shen Q Liu Z Chi Y. Possible role of *Tremella fuciformis* polysaccharide in modulating in vitro digestion of potato starch digesta. Food Chem X. (2025) 29:102759. doi: 10.1016/j.fochx.2025.102759, PMID: 40704183 PMC12284542

[82] Jin X Ruiz Beguerie J Sze DM Chan GC. *Ganoderma lucidum* (reishi mushroom) for cancer treatment. Cochrane Database Syst Rev. (2016) 2016:CD007731. doi: 10.1002/14651858.CD007731.pub3 PMC6353236 27045603

[83] Wanmuang H Leopairut J Kositchaiwat C Wananukul W Bunyaratvej S. Fatal fulminant hepatitis associated with *Ganoderma lucidum* (Lingzhi) mushroom powder. J Med Assoc Thail. (2007) 90:179–81. PMID: 17621752

[84] Kreider RB Gonzalez DE Hines K Gil A Bonilla DA. Safety of creatine supplementation: analysis of the prevalence of reported side effects in clinical trials and adverse event reports. J Int Soc Sports Nutr. (2025) 22:2488937. doi: 10.1080/15502783.2025.2488937, PMID: 40198156 PMC11983583

[85] Zeijlon R Hantelius V Wallerstedt SM Holmqvist L. Sports nutrition supplements and adverse events – a meta-epidemiological study of case reports specifically addressing causality assessment. Eur J Clin Pharmacol. (2022) 78:1–9. doi: 10.1007/s00228-021-03223-9, PMID: 34599661 PMC8724217

