# Supplementary material for: Effects of fungal supplementation on endurance, immune function, and hematological profiles in adult athletes: a systematic review and meta-analysis
Source: Front Nutr. 2025 Nov 6;12:1670416. doi: 10.3389/fnut.2025.1670416 (PMC12631420; doi:10.3389/fnut.2025.1670416)
Supplement: Supplementary file 2 [file Table_2.pdf]

Table S2. Multilingual search strategies used in CNKI and J-STAGE databases

Database	Language	Search Strategy
CNKI (China National Knowledge Infrastructure)	Chinese（中文）	AB = "运动员" AND (AB = "蘑菇" OR AB = "真菌" OR AB = "虫草" OR AB = "冬虫夏草" OR AB = "狮鬃菌" OR AB = "猴头菇" OR AB = "木耳" OR AB = "毛木耳 " OR AB = "云耳" OR AB = "银耳" OR AB = "灵芝" OR AB = "赤芝" OR AB = "云芝" OR AB = "多孔菌" OR AB = "蘑菇多糖" OR AB = "平菇" OR AB = "舞茸" OR AB = "灰树花" OR AB = "香菇" OR AB = "香蕈" OR AB = "桑黄")
J-STAGE (Japan Science and Technology Information Aggregator, Electronic)	Japanese（日文）	(キノコ OR 真菌 OR コルディセプス OR オフィオコルディセプス・シネンシス OR ヤマブシタケ OR ヘリシウム・エリナケウス OR キクラゲ OR アウリクラリア・アウリクラ OR アウリクラリア・ヘイムエル OR アウリクラリア・コルネア OR シロキクラゲ OR トレメラ・フシフォルミス OR レイシ OR ガノデルマ・ルシダム OR コリオルス・ベルシカラー OR マッシュルーム多糖 OR ヒラタケ OR プレウロトス・オストレアトゥス OR マイタケ OR グリフォラ・フロンドサ OR シイタケ OR レンチヌラ・エドデス OR フェリヌス・リンテウス) AND (運動選手 OR アスリート)
